# Food consumption and adherence to dietary guidelines among Jordanian children and adolescents

**DOI:** 10.12688/f1000research.138866.1

**Published:** 2023-09-01

**Authors:** Buthaina Alkhatib, Huda Al Hourani, Islam K. Al-Shami, Ayoub Al-Jawaldeh

**Affiliations:** 1 Department of Clinical Nutrition and Dietetics, Faculty of Applied Medical Sciences, The Hashemite University, Zarqa, Jordan; 2 Regional Office for the Eastern Mediterranean, Committee for World Health Organization, Cairo, Egypt

**Keywords:** Food consumption, discretionary calories, energy intake, MyPlate dietary guidelines, added sugars, saturated fats

## Abstract

**Background**: To evaluate the energy and macronutrient intake of Jordanian children and adolescents, and how closely they adhere to dietary recommendations.

**Methods:** This cross-sectional study used data from Jordan’s Population-based Food Consumption Survey, a household population-based study that was conducted in Jordan between 2021-2022 (561 children and adolescents 8-19 years). Dietary intake was assessed using the two non-consecutive 24-hour dietary recall method (24-h DR). The estimated food group and nutrient intakes were compared to nutritional recommendations, including MyPlate dietary guidelines.

**Results:** The prevalence of overweight/obese individuals based on body mass index (BMI) was 44%, and the average waist-to-height ratio (WHtR) was 24.7%. Compared to MyPlate dietary guidelines, children and adolescents had a higher added sugar intake (57g/day). Also, the consumption of vegetables, fruits, and dairy fell short of MyPlate dietary guidelines. The total discretionary calorie intake in children and adolescents was approximately one-third of the total energy intake.

**Conclusion:** The food consumption of Jordanian children and adolescents includes high intakes of discretionary calories, with a low intake of fruits, vegetables, and dairy products. The prevalence of overweight/obesity was elevated compared to international norms.

## Introduction

Food habits formed early in childhood are likely to follow and form the foundation of adult eating patterns. Early-life food consumption patterns may affect children’s health by increasing susceptibility to developing non-communicable chronic diseases (NCDs) in adulthood. ^1^^,^^2^ Many NCDs risk factors among children and adolescents, such as obesity, are caused by unhealthy eating behaviors and a lack of physical activity. ^3^^–^^5^

Changes in food consumption patterns in children and adolescents can be attributed to a variety of factors, including but not limited to being at school and interacting with peers during school days, the widespread availability of processed foods, availability of inexpensive food portions with family-size, eating out of home, and the ease of food delivery applications. ^6^^–^^8^

Numerous studies have examined how children and adolescents worldwide are adopting unhealthy eating habits, such as skipping meals like breakfast, ^9^^,^^10^ unhealthy snacking which is composed of energy-dense foods, ^11^^–^^13^ drinking sweetened beverages, ^14^^,^^15^ eating fast foods, ^14^^,^^16^ and consuming little fruits and vegetables. ^14^^,^^15^^,^^17^ These unhealthy habits can lead to excessive or insufficient nutrient intake, defined as ‘Nutritional status deterioration’, and have serious health consequences. ^18^ Increased risk of overweight and obesity among children and adolescents is one of these severe health problems. Childhood dietary intake plays a significant role in preventing and treating childhood overweight and obesity as it is a crucial factor in growth and development. ^19^ Obesogenic eating patterns have been extensively investigated and linked to an increased risk of becoming overweight or obese. ^20^ Among these eating patterns was the consumption of identified obesogenic foods (such as sweetened beverages, fast food, and snacks). ^21^ An investigation in Greece found that children who consume obesogenic foods are more likely to be obese. ^22^ In China, the modern dietary pattern was positively associated with future obesity in children and adolescents. ^23^ Moreover, a higher intake of vegetables in childhood has been connected with a lower chance of having mental health disorders later in life, ^24^ such as depression. Moreover, a nutritious diet can help prevent cardio-metabolic multi-morbidities, which are frequently observed in adult patients with neuropsychiatric problems. ^25^ To maintain a regular immune response, lower the risk of adverse mental health disorders and associated co- and multi-morbid conditions later in life, and maintain a regular immune response, dietary changes made as early as during childhood and adolescence represent a promising therapeutic strategy. ^26^

Over the past 40 years, the consumption of discretionary or luxury snack foods has increased globally, ^27^ and it has been linked to overweight/obesity prevalence and increasing chronic disease risk as a consequence. ^28^ Discretionary foods have been defined as food and beverages that contain added fat, sugar, and sometimes salt and are not considered necessary for a healthy diet, and contribute to excessive energy intake and displace core foods and essential nutrients. ^29^^,^^30^ Although some recommendations have been made, most dietary guidelines around the world discourage the consumption of these foods. For American children and adolescents, the discretionary calorie allowance ranges between 8% and 20% for all sex-age groups. ^31^ The recommended maximum discretionary foods and beverages for Australian children and inactive adolescents are 0-1/2 servings/day, which equals 143 kcal. ^32^

There has been little research on Jordanian children’s and adolescents’ dietary habits, and there is only sporadic data to suggest that certain food groups or individual items are consumed insufficiently or excessively. ^33^ This study aimed to 1) characterize the food consumption of Jordanian children and adolescents and 2) assess their adherence to dietary guidelines, especially MyPlate.

## Methods

### Ethical statement

This study received ethical approval from the Institutional Board Review (IRB) committee, reviewed and approved the survey protocol at The Hashemite University (No.7/13/2020/2021). Following the head of the household’s oral permission, one of the participant’s parents signed an informed consent form for children and adolescents under the age of 16. A written consent form was signed by adolescents over 16 years, and data collection began during the first visit in the presence of one of the parents.

### Design and sample selection

The current study is based on the Jordan’s Population-based Food Consumption Survey (JPFCS), which was conducted between October 2021 and March 2022. The JPFCS was created to collect dietary habits, eating patterns, and nutritional status among children, adolescents, adults, and the elderly, individuals aged eight years and older who live in Jordan. A representative sample of 701 households was invited to participate. The number of agreed-upon households to participate was 632, with a 90.2% response rate drawn randomly from Jordan’s three major regions (Central, Northern, and Southern). A total of 2,721 household members were approached, and 2,145 participants agreed to participate. The total number of children and adolescents who took part was 617 (28.7%). Children and adolescents with chronic diseases, physical disabilities, food allergies and intolerance, or cognitive impairment were excluded from the study. A study protocol has been described in detail by Al-Shami *et al.* (2023). ^34^

The current study included JPFCS children and adolescents divided into two age groups: children (8-12 years) and adolescents (13-19 years). Obtaining food intake for children under eight during the past 24 hours is challenging, especially while they are away from home (food eaten in kindergarten or daycare centers). ^34^ As a result, data reliability is higher for children aged eight and above. Outlier values for participants’ energy intake were calculated by excluding any value that increased or decreased by 2.5 standard deviations of the group mean, as outliers can have a big impact on statistical analyses, and skew the results of any hypothesis test if they are inaccurate. As a result, from 617 JPFCS participants, the final net sample size for the current analysis was 561 participants eligible. Following the head of the household’s oral permission, one of the participant’s parents signed an informed consent form for children and adolescents under the age of 16, a written consent form was signed by adolescents over 16 years, and data collection began during the first visit in the presence of one of the parents.

### Survey tool

It was announced on the university’s website about the need for nutritionists to work in data collection, and they were selected after personal interviews and based on their experience, and they were subsequently trained. The abovementioned trained nutritionists conducted a face-to-face interview with the head of the household to obtain dietary and anthropometric measurements, particularly for children (see extended data ^35^). Dietary data were collected from the children (<12 years) with parents’ help, while older participants were those who answered their dietary questions. The aforementioned data was gathered via a structured questionnaire completed during the interview. An experienced nutritionist used established methods to measure anthropometric measurements of children and adolescents, including height (cm) using Seca portable stadiometer), weight (kg) (using Tanita digital weighing scale), and waist circumference (WC) (cm) (using Seca measuring tape). Participants’ body weight and height were recorded to the nearest 0.1kg and 0.1cm. A flexible anthropometric tape was used to measure the participant’s WC while standing. Body mass index (BMI) was calculated ’as weight (kg) divided by the square of the height (m ^2^). ^36^ Moreover, the BMI-for-Age Z-Score (BAZ) was calculated. For the association of BMI-for-age with overweight and obesity, values > +1 SD represent overweight, and values > +2 SD represent obesity according to WHO reference curves. Values between +1 SD and -2 SD were considered ‘normal’. In comparison, values > -2 SD were considered ‘thinness’. The waist-to-height ratio (WHtR) was calculated by dividing the participant’s waist circumference by their height. A WHtR cutoff of ≥ 0.5 is generally accepted as a universal cutoff for obesity in children. ^37^

### Dietary assessment

Two non-consecutive 24-hour dietary recalls (24-h DR) were obtained to assess food intake on one weekday and one weekend. Furthermore, the child’s parents were asked to be present during the collection of the 24-hour DR. The first 24-h DR was collected during the interview, while the second recall was obtained via phone. Participants were asked to recall and list all foods and beverages they consumed from midnight to midnight the previous day, along with their quantity, preparation method, and most popular food brand names. A photographic food atlas was also created to simplify the estimation of consumed food portion size; this tool contains over 150 foods and composite recipes consisting of high-definition colored photos of various foods and meals commonly consumed in Jordanian diets. The survey was introduced in Arabic language; then it was translated into English before the analysis. ^35^

Three food composition databases were used to estimate nutrient intake in children and adolescents. The consumed foods collected by 24-DR were entered into a Computerized Nutrient Analysis Program: ESHA’s Food Processor ^®^, Nutrition Analysis Software (version 11:0; ESHA Research), Composition of Local Jordanian Food Dishes, ^38^ and Lebanon Food Composition Data: Traditional Dishes, Arabic Sweets, and Market Foods. ^39^ Furthermore, based on MyPlate dietary guidelines, consumed foods were classified into five food groups (grains, vegetables, fruits, protein, and dairy), with a specified number of consumed servings of each food group. According to the Essential Guide to Nutrient Requirements, published by the Institute of Medicine in the national academies press in 2019, it also was approved and recommended by the USDA Dietary Guidelines for Americans, 2020-2025, which reflects the Acceptable Macronutrients Distribution Ranges (AMDR). ^40^ The mean recommended energy intake for children and adolescents based on the MyPlate Dietary Guidelines and AMDR were 1800 and 2200, regardless of gender. ^5^ The recommended daily intake of fruits and vegetables based on the Joint FAO/WHO Expert Consultation on Diet, Nutrition and the Prevention of Chronic Diseases is a minimum of 400 g of fruits and vegetables/day; an equivalent of ≥5 servings/d. ^41^

### Statistical analysis

Analysis was conducted using SPSS software (IBM SPSS Statistics for Windows, Version 23.0. Armonk, NY: IBM Corp) ( https://www.ibm.com/products/spss-statistics). PSPP ( https://www.gnu.org/software/pspp/), a freely accessible software, is capable of running the same analysis used in this study. All analyses were stratified by gender and age group. Anthropometric characteristics were described using frequencies and percentages for categorical variables. The energy and macronutrient intake distribution was reported as means and standard deviation (Mean ± SD). An Independent sample t-test was used to compare differences between boys and girls within each age group. The normality of the distributions was assessed through the Kolmogorov-Smirnov test and Kurtosis and Skewness values. Differences were considered significant at *p* < 0.05.

## Results

### Baseline characteristics of the participants

A total of 561 Jordanian children and adolescents participated in this study, of whom 217 (38.7%) were children and 344 (61.3%) were adolescents. ^69^ The general characteristics of the study population are presented in Table 1. Although boys and girls accounted for almost equal proportions within the children group, adolescent girls accounted for the disproportionation when compared to boys within the same age group (57% vs. 43%, respectively). For the overall children group, participants who consumed dietary supplementations accounted only 6%, with no significant difference between boys and girls. A higher proportion of adolescents were using dietary supplements (any vitamins or minerals or herbs that are consumed as capsules, tablets, or drinks, instead of the diet) (12.5%), with a significant difference between both genders. Adolescent girls were found to be the most frequent group in using nutritional supplements (16.3%) when compared to boys (7.4%) from the same group ( *p* = 0.014). When compared to other regions in Jordan that were distributed according to their geographic distribution, the central regions had higher proportions of either children or adolescents of both genders (children: 61.8% of boys and 53.3% of girls; adolescents: 56.1% of boys and 62.2% of girls).

**Table 1.** Sociodemographic and anthropometric characteristics of the study population ( *n* = 561).

Variable	All (8-12 y)	Children 217 (38.7%)			All (13-19 y)	Adolescents 344 (61.3%)		
		Boys 110 (50.7%)	Girls 107 (49.3%)	*p*-value		Boys 148 (43%)	Girls 196 (57%)	*p*-value
**Geographical distribution of participants among regions**								
Center	125(57.6)	68 (61.8)	57 (53.3)	0.29	205 (59.6)	83 (56.1)	122 (62.2)	0.48
North	80 (36.9)	35 (31.8)	45 (42.1)		116 (33.7)	55 (37.2)	61 (31.1)	
South	12 (5.5)	7 (6.4)	5 (4.7)		23 (6.7)	10 (6.8)	13 (6.6)	
**Using nutritional supplements**								
Yes	13 (6.0)	4 (3.6)	9 (8.4)	0.14	43 (12.5)	11 (7.4)	32 (16.3)	**0.01**
No	204 (94)	106 (96.4)	98 (91.6)		301 (87.5)	137 (92.6)	164 (83.7)	
**BMI-for-age** **†**								
Thinness	8 (3.7)	6 (5.5)	2 (1.9)	0.24	12 (3.5)	5 (3.4)	7 (3.6)	0.82
Normal	113 (52.3)	53 (48.2)	60 (56.6)		222 (64.5	93 (62.8)	129 (65.8)	
Overweight/Obese	95 (44.0)	51 (46)	44 (41.5)		110 (32.0)	50 (33.8)	60 (30.6)	
**WHtR** **††**								
Normal	162 (75.3)	82 (74.5)	80 (76.2)	0.78	257 (74.7)	103 (70)	154 (78.6)	0.06
Abnormal	53 (24.7)	28 (25.5)	25 (23.8)		87 (25.3)	45 (30)	42 (21.4)	

	**Whole sample analysis (n = 632 households)**
**Family size (mean± SD)**	4.5±1.6
**Household monthly income**	
<500 JD	308 (49.8)
500-1000 JD	232 (37.5)
>1000 JD	79 (12.8)

* Bold *p*-value: < 0.05 considered statistically significant (2-tailed) between genders within each age group.

^†^ BMI-for-age values > +1 SD represent overweight, values > +2 SD represents obesity, values between +1 SD and -2 SD were considered “normal”, while values > -2 SD were considered “thinness”.

^††^ WHtR ≥ 0.5 is generally accepted as a universal cutoff for obesity in children and adolescents.

Findings related to the participant’s anthropometric characteristics stratified by age and gender are presented in Table 1. Most of the adolescents were classified as normal weight (64.5%), and about half of the children were also within the normal weight category (52.3%). Without significant differences between both genders among each age group; children boys had the highest overweight/obesity prevalence (46.3%), followed by girls in the same age group (41.5%), then adolescent boys (33.8%), and the lowest prevalence was for adolescent girls (30.6%). About a quarter of the children’s boys had abnormal WHtR (25.5%), and 23.8% of the same group of girls too; however, 30.4% and 21.4% of adolescent boys and girls, respectively, were also classified as having abnormal WHtR, with no significant differences. On the other hand, based on the household analysis, there were 4.5 people per family on average. Additionally, nearly 50% of the sample under study (JPFCS original sample) stated that their household’s overall income is less than 500 JD.

### Graphic representation of adherence to MyPlate dietary guidelines

**Figure 1.**
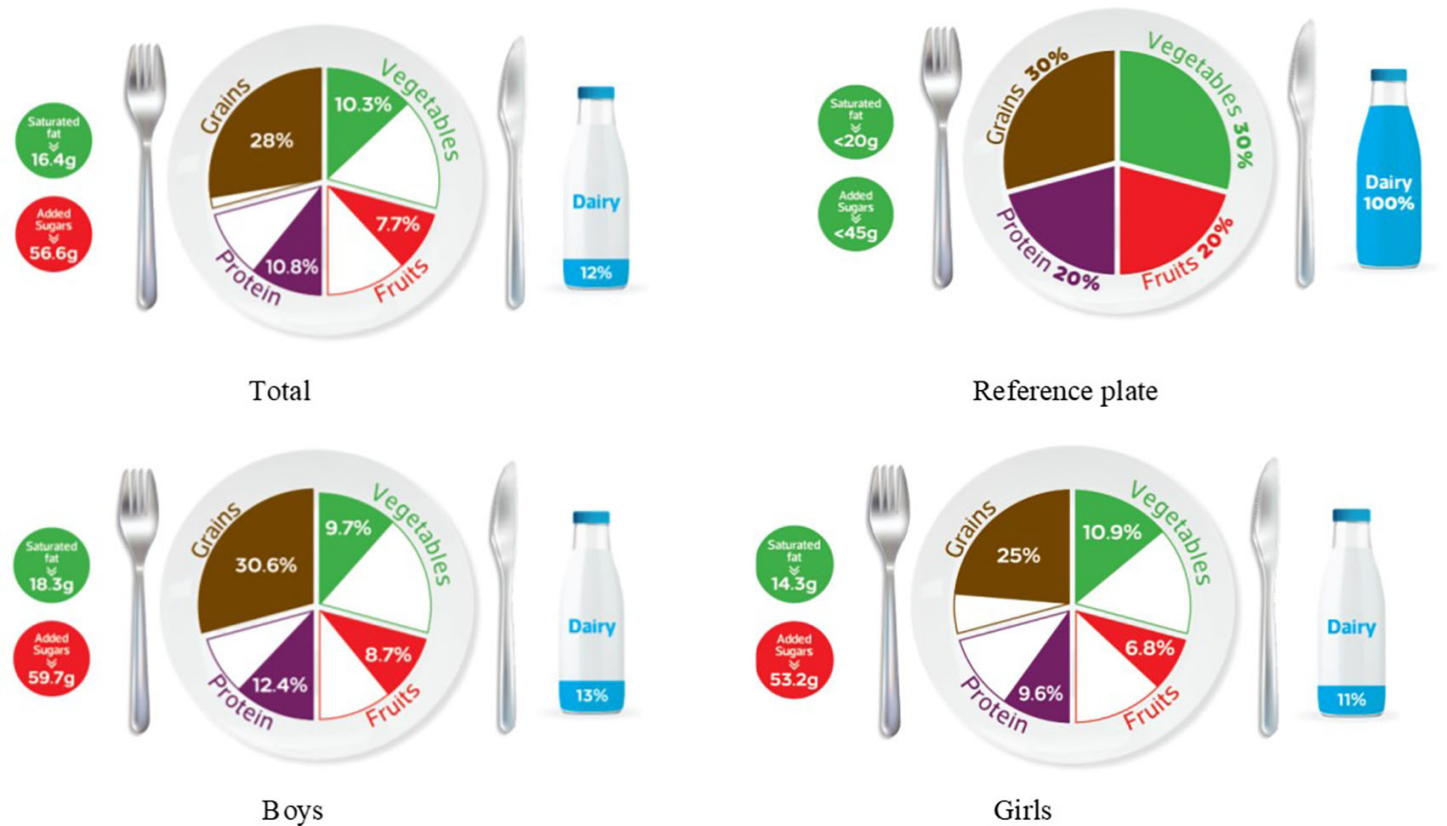
Compliance of the mean habitual food consumption with the MyPlate dietary guidlines in children.

**Figure 2.**
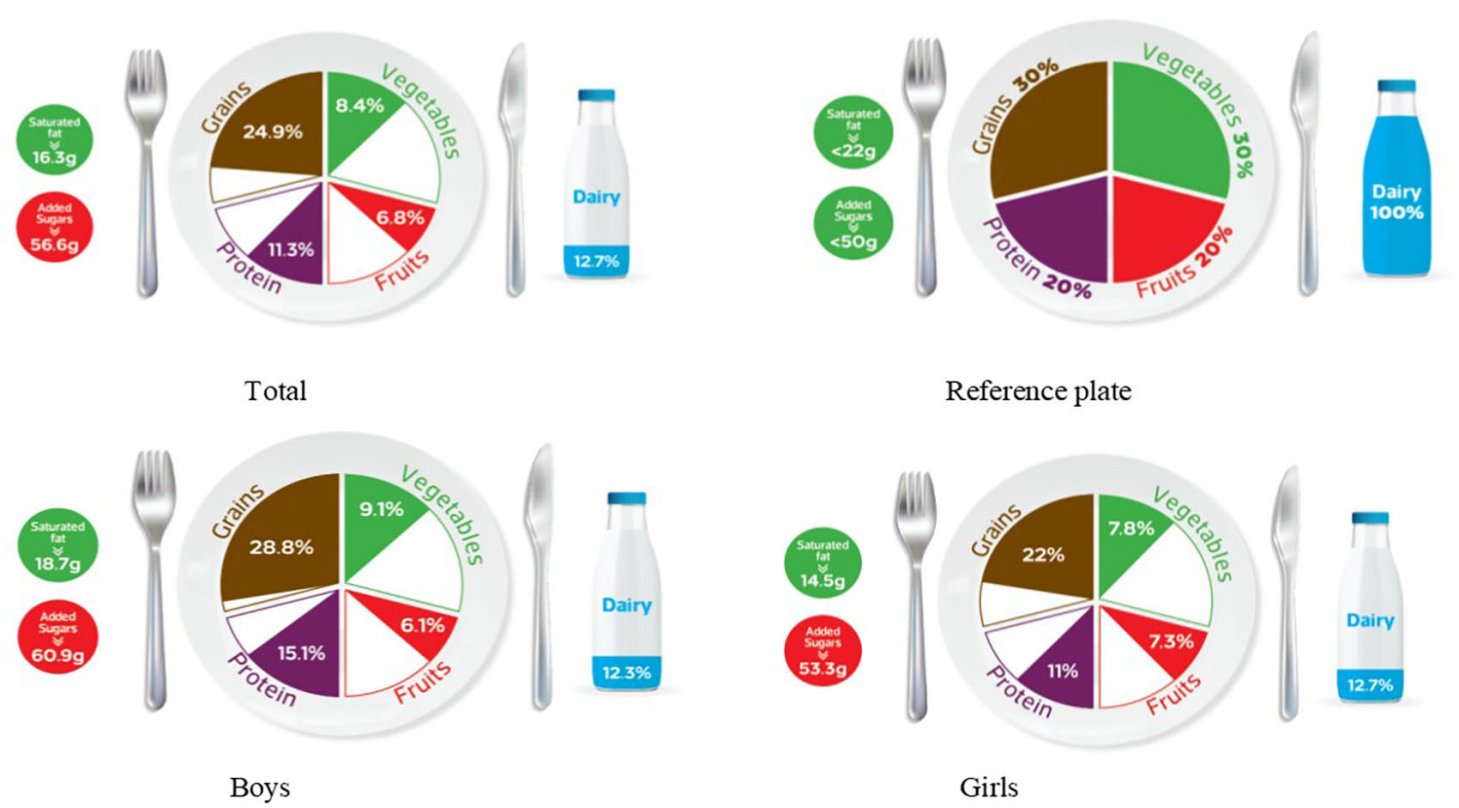
Compliance of the mean habitual food consumption with the MyPlate dietary guidlines in adolescents.

Table 2 categorizes the most common food items consumed by Jordanian children and adolescents into five food groups, as well as the recommended number of servings for each food group for both age groups, based on 1800 kcal needs for children and 2200 kcal needs for adolescents. The reference MyPlate dietary guidelines image was presented for children in Figure 1 and adolescents in Figure 2.

**Table 2.** Daily recommendations of the food groups according to MyPlate dietary guidelines within calorie allowance.

	Foods Included in the Analyses	Serving Size	Recommended Daily intake	
			Children	Adolescents
**Food group**				
Grains	Thin white or whole wheat Kmaj bread, thick Kmaj white or whole wheat bread, burger bun, Sammon (Sammoli or Hamam, a pan white French-like bread), shrak bread (non-pocket, one-layered flatbread). Rice and rice in dishes, macaroni, cornflakes	One ounce of bread (any type), ½ cup cooked rice or macaroni, ½ cup cornflakes	6 oz	7 oz
Vegetables	Cooked vegetables such as green beans, okra, and spinach. Raw vegetables such as tomatoes, cucumber, lettuce, and cabbage	½ cup cooked vegetables or 1 cup raw vegetables.	2.5 Cups	3 Cups
Fruits	Fresh fruits such as apples, bananas, and oranges. 100% fruit juices, dried and canned fruits	One small fresh fruit, ½ cup 100% fruit juice, two tablespoons dried fruits, ½ cup canned fruits.	1.5 Cups	2 Cups
Dairy	Milk, yogurt, white cheese in brine, spreadable processed cheese, cheddar-flavored processed cheese, and labneh (condensed salted yogurt)	One cup of milk yogurt, 45 gm cheese, and 2 tablespoons of labaneh.	3 Cups	3 Cups
Protein	Lamb and veal meat, chicken, fish, tuna and sardine, sausages, boiled and fried eggs, and any cooked vital organs of chicken, lamb, and veal (liver, heart, kidneys, tongue, …)	2-3 oz of cooked lamb, veal, chicken, or fish, 1 small can of tuna or sardine, 2 eggs,	5 oz	6 oz
**Foods to limit**				
Added sugars	Beverages like: Carbonated beverages, canned juices (fruit drinks), any sweetened ready-to-use hot drink, and sweetened tea and coffee; Sweets and desserts: Sugar, jam and honey, ice cream, Jell-O, pudding, custard, chocolate, cake, Arabic sweets (baklava, harissa)	One teaspoon of sugar, 1 Tablespoon jam or honey, ½ cup of ice cream, ½ cup of Jell-O or pudding or custard, 1 ounce of chocolate, 2-ounce cake, 1 ounce of Arabic sweets	<45 g	<50 g
Saturated fat	Found naturally in meat and its substitute, in any animal organ, as well as in whole and reduced fat milk, in whole fat dairy products, in eggs, and many restaurant-type foods, in butter, ghee, lard, in any animal extracted fat	One teaspoon of butter, ghee, lard	<20 g	<22 g

1 oz = 28.5 g; 1 cup = 24 0mL.

In children, the entire group consumes less than the recommended number of servings from all food groups, as well as girls of the same age group. On the other hand, children’s boys almost consumed the recommended amount of grains (30.6% vs. 30% of the standard plate), while all other food groups were lower than the recommended number of servings, as shown in Figure 1. Mismatching between real and recommended intake based on the standard plate has been indicated. As shown in Figure 1, a higher gap has been found among children girls’ intake of dairy, protein, and fruits (11%, 9.6%, and 6.8%, respectively) when compared to boys from the same age group (13%, 12.4%, and 8.7%; respectively) ( Figure 1). Furthermore, Jordanian adolescents overall or according to gender differences consumed less than the recommended serving number of each food group ( Figure 2), based on 2200 kcal/day as the average total energy requirement ( Table 2). In girls, the actual intakes of grains, fruits, vegetables, and proteins were 22%, 7.3%, 7.8%, and 11%, respectively, which did not agree with the recommended reference plate. This discrepancy was also observed in boys with their consumption of fruit and vegetables (6.1% and 9.1%, respectively). However, there appears to be less mismatch in grain and protein intake (28.8% and 15.1%, respectively) ( Figure 2). It’s interesting to note that both age groups showed the same outcome in terms of gender differences because there was a significant difference in their intake of grains and proteins ( *p*-value < 0.05) among all the food groups.

### Energy and macronutrient intakes among the study population

The mean daily energy and macronutrient intake of the study population, stratified by age and gender, is shown in Table 3. In the children’s group, the mean total energy intake was: 1796.4 ± 557.1 kcal/day, with a significant difference between boys and girls; the mean total energy intake for boys was 1950 kcal/day compared to 1638 kcal/day for girls ( *p* < 0.001). Similar results were shown in the adolescent group, with a significantly higher mean energy intake in boys compared to girls (2116.3 kcal/day vs. 1688.5 kcal/day) ( *p* < 0.001), while the average total intake of the group was 1872. 6 ± 579.1 kcal/day.

**Table 3.** Mean daily intakes of energy (kilocalories) and macronutrients stratified by age group and gender.

	Total (8-12 y)	Children			Total (13-19 y)	Adolescents		
		Boys	Girls	*p-*value		Boys	Girls	*p*-value
**Energy and macronutrients**								
**Total Energy intake (EI) (kcal)**	1796.4 ± 557.09	1950.01 ± 602.39	1638 ± 457.83	**<0.001**	1872.57 ± 579.06	2116.34 ± 600.39	1688.5 ± 488.8	**<0.001**
**Total carbohydrates (g)**	230.82 ± 73.95	247.83 ± 81.84	213.33 ± 60.38	**0.001**	234.47 ± 75.26	260.96 ± 76.41	214.47 ± 68.02	**<0.001**
**% From the total EI**	51.75 ± 6.61	51.11 ± 7.11	52.4 ± 6.02	0.154	50.37 ± 7.34	49.72 ± 6.91	50.86 ± 7.63	0.153
**Total protein (g)**	59.19 ± 20.82	65.43 ± 23.61	52.77 ± 15.11	**<0.001**	67.72 ± 25.92	78.99 ± 28.54	59.21 ± 19.96	**<0.001**
**% From the total EI**	13.24 ± 2.45	13.046 ± 2.53	13.01 ± 2.34	0.184	14.58 ± 3.69	15.01 ± 3.69	14.24 ± 3.66	0.056
**Total fat (g)**	73.32 ± 27.85	80.17 ± 29.29	66.27 ± 24.49	**<0.001**	75.79 ± 29.45	86.01 ± 32.02	68.08 ± 24.77	**<0.001**
**% From the total EI**	36.33 ± 6.05	36.73 ± 6.23	35.93 ± 5.85	0.333	36.05 ± 6.6	36.1 ± 6.44	36.02 ± 6.73	0.915
**Types of fat**								
**Polyunsaturated fats (g)**	10.91 ± 7.25	12.24 ± 8.09	9.54 ± 6.01	**0.006**	10.86 ± 7.05	12.69 ± 8.07	9.47 ± 5.81	**<0.001**
**% From the total EI**	5.26 ± 2.58	5.42 ± 2.73	5.09 ± 2.43	0.354	5.11 ± 2.54	5.25 ± 2.54	5 ± 2.55	0.354
**Monounsaturated fats (g)**	19.29 ± 10.28	21.32 ± 11.02	17.21 ± 9.04	**0.003**	19.79 ± 11.36	23.24 ± 12.59	17.17 ± 9.57	**<0.001**
**% From the total EI**	9.55 ± 3.98	9.76 ± 4.21	9.33 ± 3.74	0.434	9.4 ± 4.17	9.83 ± 4.37	9.09 ± 3.99	0.105
**Saturated fats (g)**	16.35 ± 7.47	18.33 ± 7.46	14.31 ± 6.93	**<0.001**	16.32 ± 8.15	18.69 ± 9.09	14.53 ± 6.85	**<0.001**
**% From the total EI**	8.16 ± 2.66	8.52 ± 2.64	7.8 ± 2.64	**0.047**	7.78 ± 2.78	7.87 ± 2.7	7.7 ± 2.85	0.603
**Trans fat (g)**	0.5 ± 0.8	0.52 ± 0.81	0.47 ± 0.8	0.64	0.6 ± 1.2	0.81 ± 1.51	0.45 ± 0.88	**0.006**
**% From the total EI**	0.25 ± 0.43	0.25 ± 0.42	0.26 ± 0.43	0.863	0.27 ± 0.48	0.3 ± 0.52	0.24 ± 0.46	0.254
**Omega-3 Fatty acids (g)**	0.57 ± 0.5	0.64 ± 0.53	0.48 ± 0.45	**0.018**	0.63 ± 0.5	0.73 ± 0.58	0.56 ± 0.49	**0.004**
**Omega-6 Fatty acids (g)**	5.96 ± 5.24	6.86 ± 5.7	5.03 ± 4.57	**0.01**	6.12 ± 5.12	6.89 ± 5.73	5.53 ± 4.53	**0.015**
**Cholesterol (mg)**	133.22 ± 93.74	147.76 ± 100.9	118.28 ± 83.61	**0.02**	168.07 ± 150.1	218.51 ± 192.64	129.8 ± 90.47	**<0.001**

MUFA: monounsaturated fatty acids; PUFA: polyunsaturated fatty acids; FA: fatty acids; EI: energy intake.

* *p*-value < 0.05 is considered statistically significant (2-tailed).

Table 3 further examines the various types of fat consumed; the mean saturated fat (SFA) intake was nearly equal for both age groups and rounded to 16 g/day ( Table 3), which was within an acceptable amount when compared to the MyPlate dietary guidelines allowed amounts of 20 g/day and 22 g/day for children and adolescents, respectively ( Table 2, Figure 1, and Figure 2). Boys of both ages consumed significantly more saturated fats than girls of the same age (children: 18.3 g/day vs. 14.3 g/day, and adolescents: 18.7 g/day vs. 14.5 g/day, for boys and girls, respectively) ( *p* < 0.001) ( Table 4).

**Table 4.** Mean and Standard deviation of discretionary calorie intake among the study population.

	Total (8-12 y)	Children			Total (13-19 y)	Adolescents		
		Boys	Girls	*p-*value		Boys	Girls	*p*-value
**Discretionary calories**								
**Added sugars (g)**	56.5 ± 31.7	59.68 ± 36.39	53.22 ± 25.76	0.133	56.55 ± 28.58	60.87 ± 28.88	53.28 ± 27.97	**0.015**
**% From the total EI**	12.95 ± 6.06	12.52 ± 6.01	13.39 ± 6.1	0.286	12.31 ± 5.58	11.83 ± 5.38	12.68 ± 5.72	0.166
**Added fats (g)**	36.06 ± 18.21	39.33 ± 18.87	32.7 ± 16.95	**0.007**	34.66 ± 20.15	38.92 ± 22.3	31.42 ± 17.73	**0.001**
**% From the total EI**	17.7 ± 6.26	17.89 ± 6.09	17.51 ± 6.45	0.654	16.31 ± 7.3	16.08 ± 7.12	16.49 ± 7.44	0.612
**Total Discretionary calories intake**								
**% From the total EI**	30.65 ± 7.99	30.41 ± 7.99	30.9 ± 8.04	0.648	28.63 ± 9.01	27.92 ± 8.86	29.16 ± 9.1	0.205

* *p*-value < 0.05 is considered statistically significant (2-tailed). EI: energy intake.

Moving in sequence, the mean intake of polyunsaturated (PUFA) and monounsaturated fats (MUFA) was nearly equal in both age groups; however, boys in both age groups consumed significantly more than girls ( *p* < 0.05) ( Table 3). Trans fat (TFA) intake was significantly higher in adolescent boys than in girls (0.81 g/day vs. 0.45 g/day) ( *p* = 0.006). Irrespective of gender-specific differences, the mean cholesterol intake of the adolescents was higher than that of the children’s group (168.1 ± 150.1 mg/day vs. 133.2 ± 93.7 mg/day; respectively). Also, mean cholesterol intake was significantly higher in boys than in girls in the child group (148 mg/day vs. 118 mg/day ( *p* = 0.02) and in adolescents (219 mg/day for boys vs. 130 mg/day for girls) ( *p* < 0.001). In addition, the intake of omega-3 and omega-6 fatty acids was significantly higher in boys than in girls in both age groups ( Table 3).

### Consumption of discretionary calories

According to the MyPlate dietary guidelines objectives that make every bite count and choose options full of nutrients, some foods and beverages were recommended to be explicitly limited; added sugars and saturated fats/added fat. Foods that were analyzed as added sugars and saturated fats were listed in Table 2, as well as their typical serving sizes, and the maximum allowed amount to consume for each age group were presented in the same table.

Age differences in mean intake from added sugar were almost equal to 57 g/day, and this means higher than MyPlate dietary guidelines allowed amounts which were <45 g/day and <50 g/day for children and adolescents, respectively ( Table 4, Figure 1, and Figure 2). Although no significant difference between boys and girls was found within the children group, adolescent boys significantly consumed higher amounts of added sugars when compared to girls from the same group (60.87 g/day vs. 53.28 g/day, respectively) ( *p* = 0.015). Moreover, children and adolescent boys consumed significantly higher amounts of total added fats than girls (39.33 g/day, 38.92 g/day vs. 32.7 g/day, and 31.42 g/day ( *p* = 0.007; 0.001; respectively) ( Table 4). For children and adolescents, total discretionary calorie intake (added sugar and fat) accounted for approximately 30.7% and 28.6% of total energy intake, respectively; which is considered higher than the recommendations (<10% of total energy intake).

## Discussion

The food consumption of Jordanian children and adolescents has never been studied before. Furthermore, the COVID-19 pandemic is a significant contributor to the shift in dietary intake globally toward obesogenic food choices, besides decreased physical activity, increased screen time, and leading to weight gain. ^42^^,^^43^

The present study’s findings showed that the overall prevalence of generalized overweight/obesity among the studied sample was 36.6%. The prevalence of overweight/obesity was higher in children than in adolescents (44.3% vs. 32%). About 1/5 to 1/4 of participants from all age groups were classified as overweight. Globally, overweight and obesity have increased dramatically among children and adolescents aged five to 19, from 4% in 1975 to just over 18% in 2016. ^44^ In Jordan, the image was not dissimilar to other countries worldwide. Zayed *et al.* (2016) reported that analysis by gender showed that obesity was more prevalent among Jordanian males, with 18.9% obese compared to 11.2% of females. ^45^ Jordanian obesity prevalence and its related gender patterns appear consistent with what has been reported in the literature.

Another obesity index, WHtR, revealed that nearly a quarter of the children’s boys (25.5%) and 23.8% of the same group of girls had abnormal WHtR; however, 30.4% and 21.4% of adolescent boys and girls, respectively, were also classified as having abnormal WHtR. WHtR was not used to assess abdominal obesity in Jordanian children and adolescents earlier. A few studies on WHtR as an indicator of obesity in the Eastern Mediterranean region reported similar results; in the United Arab Emirates (UAE), 27.2% of subjects had abnormal WHtR, ^46^ and in Lebanon, 38.3% had abnormal WHtR. ^47^

*Obesity as a result of diet has been extensively studied. Fast food and sugar-sweetened beverages are directly linked to the childhood obesity epidemic. The initial findings on the prevalence of obesity were primarily related to food intake.* This study found that adolescents engaged in eating behaviors associated with an increased risk of being overweight and obese.

The MyPlate dietary guidelines were created in 2011, indicating how much of the plate should be allocated to protein foods, grains, fruits, and vegetables. The current study’s findings revealed that children’s girls consumed fewer than the recommended number of servings from all MyPlate dietary guidelines groups. On the other hand, children’s boys nearly consumed the recommended amount of grains (30.6% vs. 30% of the plate), while all other food groups consumed less than the recommended number of servings. However, adolescents’ intake was off-balance in all recommended servings of MyPlate dietary guidelines, regardless of gender.

Compared with studies in the Eastern Mediterranean countries, 28% of Emirati adolescents met the recommended daily fruit and vegetable intake, with significantly more males than females. ^48^ A multinational comparison study of eleven Eastern Mediterranean countries revealed that 19.4% of adolescents reported consuming fruits and vegetables ≥5 times/day. ^49^ In Iran, a study found that only 30.3% and 34.6% of adolescents consumed the recommended amount of fruit and vegetables, respectively. ^50^ In Egypt, 45.9% of children and adolescents have fruits and vegetables 1-6 times daily. ^51^ In the UAE, around 50% of school-age adolescents had less than five servings of fruits and vegetables. ^46^ Similar findings were reported among Saudi children, with 69% and 71% of the samples not consuming fruits and vegetables daily. ^52^ Furthermore, a review of the dietary patterns and nutrient intakes of children (5-10 years) and adolescents (10-19 years) in the Eastern Mediterranean Regions revealed that their diet was poor, with fruits, vegetables, and dairy products. ^53^ According to current study findings and studies from Eastern Mediterranean countries, Western countries did not achieve better results in this area. In Belgium, adolescents were the least likely to follow food-based dietary recommendations compared to the youngest children and adults. ^37^ As well as, a study in 33 countries from European and North America reported that a large proportion of adolescents do not eat fruit and vegetables daily. ^54^

Although most studies reported low consumption of fruits and vegetables among children and adolescents worldwide, it is important to note that the methods used to assess their intake vary; some studies rely on self-reporting food frequency, while others rely on face-to-face 24-DR.

Although the current study’s findings showed a low consumption of fruits, vegetables, and dairy groups, the mean energy intake was within the normal range of requirements for age and gender. The mean total energy intake among the children was 1796.4 ± 557.09 kcal/day, with a significant difference between boys and girls. Similar findings were shown among the adolescent group; a significantly higher mean energy intake among boys compared to girls (2116.34 kcal/day vs. 1688.5 kcal/day; respectively) ( *p* < 0.001), while the average overall group intake was 1872.57± 579.06 kcal/day.

The present findings indicated that means of macronutrient percentages were found to comply with the AMDRs for both carbohydrates and protein. Fat was high among Jordanian children and adolescents (36.33±6.05 % and adolescents 36.05±6.6%; AMDRs for both groups: 25-35%). Similar results were observed in a previous study conducted among Jordanian adolescents aged 14-18 with a fat intake of around 37% of EI. ^55^ Such findings are comparable to those reported in other countries in the region, including Lebanon (39.2% of EI for 4-13 years), ^56^ and Palestine (39.8% of EI for boys 11-16 years). ^57^ The picture appears similar to what has been reported in Western countries including Spain, where about 43.4% of boys and 46.9% of girls between 4 and 17 years exceeded the AMDR in total fat intake, ^58^ while the mean contribution of fats to EI was 36.9% and 37.2% in Italian boys and girls; respectively (teenagers: 10-17.9 years). ^59^ Remarkably, the results of our study were consistent with those of other studies, showing that the pattern of energy and macronutrient intake has become a popular choice for trendy styles among children and adolescents worldwide.

Discretionary calories, including added fat and sugar, are unnecessary for health. These foods contribute excess energy to the diet by displacing nutritious foods and should be consumed in moderation. The total intake of discretionary calories in the current study exceeded the recommendations for both age groups by three times (30.7% and 28.6% of total EI for children and adolescents, respectively), considering that the recommendation is <10% of the total EI. ^60^ These results are comparable to those of other countries worldwide. Discretionary foods and beverages accounted for more than a third of the daily energy intake of Australian children aged 2 to 18. ^32^ The same findings were found in Pakistan, where adolescents consumed 31.3% of their energy needs from discretionary foods. ^61^ On average, about 14% of total EI in American children and adolescents was from added sugars. ^62^

Despite adequate carbohydrate intake, the average intake of added sugars was nearly 57 g/day (about 13% and 12% of total EI for children and adolescents, respectively), and these averages were higher than the levels allowed by the MyPlate dietary guidelines and WHO guidelines. These findings might be related to the frequent consumption of sweet snacks, including cakes, pastries, and sweets/chocolates. Adolescents in some Middle Eastern countries tuned increased daily consumption of sweets and desserts, such as in Sudan and Kuwait. ^53^^,^^63^ Consistently, the German Child and Adolescent Study results were no better than the present results, reporting that 50% of carbohydrate intake came from added sugars (23.5%) and total sugars, (28.1%). ^64^ In addition, the percentage of energy contribution from added sugars in US children and adolescents was 14.3% at 2-8 years and 16.2% at 9-18 years, while the primary sources are sweetened beverages, candy, and sweet baked goods. ^65^ The main sources of added sugars in the present study were sweetened beverages such as carbonated beverages, canned juices (fruit beverages), many types of sweetened ready-to-drink beverages, and sweetened tea; chocolate, cakes, as well as various kinds of Arabic sweets such as baklava, as well as Western desserts.

Contrary to previously published studies, saturated fats mean intake in the present findings was almost equal for both age groups and rounded at 16 g/day; it was within an acceptable amount compared to the MyPlate dietary guidelines allowed amounts. Notably, boys from both age groups consumed significantly higher amounts of cholesterol, saturated, polyunsaturated, and monounsaturated-fatty acids than girls from the same age group. Many Middle Eastern countries, including Lebanon, ^56^ Iran, ^50^ Palestine, ^57^ and Saudi Arabia, ^66^ had average saturated fat intakes (10.7%,10.3%, 10.3%, and 11.3%, respectively) that exceeded the recommendations (AMDR upper limit for saturated fat is 10% of total EI). A similar pattern of results was obtained in Western countries such as Italy (11.5%) the UK (13.2%), France (15%), and Spain (12.3%). ^58^^,^^59^^,^^67^^,^^68^

Some issues must be addressed to clarify the findings of this study. The large representative sample from a population-based study, the careful data quality control methods by reducing dietary intake misreporting, evaluating overweight and obesity by using WHtR (indicating central obesity) rather than BMI (indicating overall obesity), and the repaint of the specific MyPlate dietary guidelines for all age groups were major strengths of our study. One of the current study’s limitations was that it was cross-sectional, which hindered the observation of causal effects. Furthermore, there is a lack of evaluation of physical activity, nutrition-related blood tests, and blood pressure.

The findings of this study make several noteworthy contributions in depicting the food consumption patterns and food and nutrient intakes following the COVID-19 pandemic and its consequences; the disease’s effect occurred in two stages: first, during the lockdown, and second, afterward. An enormous amount of scientific literature focuses primarily on the negative changes in food habits, food consumption quality and quantity, body weight, and physical activity in people of all ages, particularly children and adolescents. Jordan experienced lockdowns and school closings like any other country, eventually replaced by e-learning. These negative changes in children and adolescents could last longer until adulthood and increase NCDs’ prevalence.

Some issues must be addressed to clarify the findings of this study. The large representative sample from a population-based study, the careful data quality control methods by reducing dietary intake misreporting, evaluating overweight and obesity by using WHtR (indicating central obesity) rather than BMI (indicating overall obesity), and the repaint of the specific MyPlate dietary guidelines for all age groups were major strengths of our study. One of the current study’s limitations was that it was cross-sectional, which hindered the observation of causal effects. Furthermore, there is a lack of evaluation of physical activity, nutrition-related blood tests, and blood pressure.

### Implications for research and practice

It appears that fat and added sugar consumption must be controlled; policymakers and health professionals must take appropriate steps to address this issue. It is recommended that families, children, and adolescents receive nutrition education about the components of MyPlate dietary guidelines and the adverse health effects of obesogenic diets. Implementing community education programs about healthy eating habits requires cooperation between governmental and non-governmental organizations.

## Conclusions

In conclusion, the prevalence of overweight/obesity approached about half of children, and a third of adolescents were classified as overweight/obese. On the other hand, about a quarter of the children and adolescents had abnormal WHtR. Notably, children and adolescents mismatched the intake of all recommended servings of MyPlate dietary guidelines (except grain intake in ’boys’ children). Despite the mean energy intake being within the estimated recommendations, children and adolescents consumed a slightly higher fat proportion than the AMDRs. Noticeably, the mean intake of added sugar was higher than MyPlate dietary guidelines allowed amounts, while the saturated fats mean intake was within an acceptable MyPlate dietary guidelines amount for both groups. Moreover, about 1/3 of energy consumed was from discretionary calories. Remarkably, boys consumed higher amounts of cholesterol, saturated, polyunsaturated, and monounsaturated fats than girls.

## Data Availability

Zenodo: Food consumption and adherence to dietary guidelines among Jordanian children and adolescents. https://doi.org/10.5281/zenodo.8159227. ^69^ The project contains the following underlying data:
• SPSS JPFCS children and Teenagers 2023. sav. (Anonymised participant’s data used in this study). Zenodo: Food consumption and adherence to dietary guidelines among Jordanian children and adolescents. https://doi.org/10.5281/zenodo.8240928. ^35^ This project contains the following extended data:
• A study of the rate of food consumption using 24-hour memory for a sample of Jordanian society.docx (English version of data collection survey including two sheets for 24-hour recall). •
دراسة معدل استهلاك الطعام باستخدام التذكر ل 24 ساعة لعينة من المجتمع الأردني النسخة العربية .docx. (Arabic version of data collection survey including two sheets for 24-hour recall). Data are available under the terms of the Creative Commons Attribution 4.0 International license (CC-BY 4.0)
