# Supplementary material for: Food consumption and adherence to dietary guidelines among Jordanian children and adolescents
Source: F1000Res. 2025 Feb 4;12:1094. Originally published 2023 Sep 1. [Version 4] doi: 10.12688/f1000research.138866.4 (PMC11409655; doi:10.12688/f1000research.138866.4)
Supplement: Peer review history

# Reviewer report: Reviewer response for version 4

**DOI:** 10.5256/f1000research.176995.r364514

**Published:** 2025-02-19

**Authors:** Sarah Ibrahim

**Affiliations:** 1 Human Anatomy and Embryology Department, Tanta University, Tanta, Gharbia Governorate, Egypt

Upon reviewing the manuscript, I have no further edits/feedback. The comments have been addressed.

Is the work clearly and accurately presented and does it cite the current literature?

Yes

If applicable, is the statistical analysis and its interpretation appropriate?

Yes

Are all the source data underlying the results available to ensure full reproducibility?

Yes

Is the study design appropriate and is the work technically sound?

Yes

Are the conclusions drawn adequately supported by the results?

Yes

Are sufficient details of methods and analysis provided to allow replication by others?

Yes

Reviewer Expertise:

NA

I confirm that I have read this submission and believe that I have an appropriate level of expertise to confirm that it is of an acceptable scientific standard.

# Reviewer report: Reviewer response for version 3

**DOI:** 10.5256/f1000research.171932.r338455

**Published:** 2024-12-03

**Authors:** Fatima Al Zahraa Chokor

**Affiliations:** 1 Qatar University, Doha, Doha, Qatar

The article investigates the dietary habits and nutritional status of Jordanian children and adolescents in relation to MyPlate dietary guidelines. The study employs data from the Jordan Population-based Food Consumption Survey (JPFCS) and analyzes dietary intake, anthropometric measurements, and adherence to dietary guidelines. The findings reveal significant deviations from recommended dietary patterns, particularly concerning low intake of fruits, vegetables, and dairy, excessive fat and added sugar consumption, and a high prevalence of overweight and obesity. The study underscores the necessity for nutrition education and policy interventions to promote healthier eating habits among Jordanian youth.

**Introduction:**

- The introduction generally flows well, moving from the broader context of childhood eating habits to the specific problem of obesity and dietary patterns in Jordan. However, the transition between general global concerns and Jordan-specific issues could be smoother. For example, the mention of Jordanian obesity prevalence as a separate paragraph of one sentence in lines 5-6 is abrupt and could benefit from a brief segue that connects global trends to the local context.
- It would be also good to discuss the long-term societal and economic implications of the increasing trends of obesity in Jordan and thus highlighting the benefits of early intervention, especially in this age group to frame the study’s relevance.
- Very little in the literature was cited from Jordan, this warrants discussion of studies from Jordan or at least from the region (regional perspective) which would provide a stronger foundation for the study. Where does Jordan stand with the nutrition transition occurring in the EMR?
- Little is mentioned on micronutrient deficiencies which is one of the objectives
- It is good to provide a brief rationale for using the MyPlate guidelines and their relevance to Jordanian dietary habits
- You have presented and evaluated dietary intakes in the study but this has not been mentioned as part of the objectives
- It would be better to reframe the objectives in a way where they can inform future interventions or policies or at least describe how this study would contribute to solving the problem of obesity or informing public health interventions

**Methods: **

- Rewrite: elderly, Jordanian individuals
- Rewrite: with a 90.2% response rate drawn simple random sampling from Jordan’s three major regions (Central, Northern, and Southern).
- Provide a more detailed explanation of how dietary data were standardized and verified for accuracy.
- Were dietary intakes normally distributed? If yes, report that in statistical analysis. If not, medians have to be reported and non-parametric tests have to be used.
- Outlier values for participants’ energy intake… has to be written under statistical analysis section
- This sentence is written under ‘Design and sample selection’ and it is already mentioned in ‘Ethical statement’: Following the head of the household’s oral permission, one of the participant’s parents signed an informed consent form for children and adolescents under 16. A written consent form was signed by adolescents over 16 years, and data collection began during the first visit in the presence of one of the parents.
- Sample size calculations not presented
- Instead of having a section for Survey Tool and another section for Dietary assessment, better to have the write-up divided into ‘anthropometric assessment’ and ‘Dietary intake assessment’
- Has there been missing data?
- Reference needed for WHO reference curves for BMI-for-age categorization
- How were households selected? The sampling strategy lacks sufficient detail.
- What is the eligibility criteria?
- It has not been mentioned how sample characteristics were collected? Income, family size, etc…
- Has there been data collected on parents’ education and employment?
- How was height collected? With or without shoes?
- How many times were anthropometric measurements done?
- What about foods consumed at school? How was this data collected?
- How were composite and mixed recipes (mixed dishes) categorized under MyPlate food groups?
- Has the average of dietary intake been calculated or the usual intake?
- This is not clear: According to the Essential Guide to Nutrient Requirements, published by the Institute of Medicine in the National Academies Press in 2019, it also was approved and recommended by the USDA Dietary Guidelines for Americans, 2020-2025, which reflects the Acceptable Macronutrients Distribution Ranges (AMDR).
- How were food groups intakes compared to dietary recommendations? Elaboration needed in the methods section
- Cite table 2 in the methods
- How were the differences in the proportions of Sociodemographic and anthropometric characteristics compared between the two sexed in each age group? Mention the test name in statistical analysis

**Results**

- Rewrite in a clearer manner: When compared to other regions in Jordan that were distributed according to their geographic distribution, the central areas had higher proportions of either children or adolescents of both sexes (children: 61.8% of boys and 53.3% of girls; adolescents: 56.1% of boys and 62.2% of girls).
- Table 1: correct percent of overweight/obesity for children boys
- Include as a footnote how was p-value obtained in Table 1, 3, and 4
- Since there is no consensus on the appropriate cut points of WHtR in pediatric populations, better to replace normal/abnormal in table 1 with <0.5 versus ≥0.5. Same applies for in-text interpretation.
- Add reference for recommendations presented in Table 2
- There are minor formatting inconsistencies, such as the use of parentheses for p-values and multiple numerical data points in a single sentence.
- Results need to better written for clarity: example: In children, the entire group consumes less than the recommended number of servings from all food groups and girls of the same age group. What do you mean by and girls of the same age group?
- Not an appropriate term to use: children’s boys almost finished the recommended … replace by: Boys came close to meeting the recommended servings for grains, achieving 30.6% of the standard plate recommendation of 30%.
- When stating: higher gap has been found among children girls’ intake of dairy, protein, and fruits (11%, 9.6%, and 6.8%, respectively) when compared to boys from the same age group (13%, 12.4%, and 8.7%; respectively), differences in compliance shall be tested for statistical significance.
- Replace: which did not agree with did not meet.
- Overall, the results section could be significantly improved by utilizing more precise and professional language to enhance clarity and readability.

**Discussion**:

- The discussion can benefit from starting by summarizing the study’s objectives (e.g., characterizing dietary patterns and adherence to guidelines) and explicitly linking the results to these objectives.
- The discussion includes a detailed elaboration on the impact of COVID-19 on dietary patterns and physical activity, but this is not mentioned at all in the introduction, nor is there any indication that data on post-pandemic changes in dietary patterns were collected. This inconsistency is problematic as it introduces a topic in the discussion that was neither contextualized nor justified earlier in the article. Additionally, without specific data related to the pandemic's effects, the inclusion of COVID-19 as a contributing factor appears speculative and unsupported by the study's findings.
- The discussion restates many results without fully interpreting their significance or exploring potential underlying causes. Example when comparing prevalence of high WHtR, prevalence of overweight and obesity, compliance to food groups recommendations. This section lacks discussion of implications and a forward-looking perspective on how the study could inform policy or future research.
- What is meant by generalized overweight and obesity?
- There is little exploration of sociocultural or regional factors specific to Jordan. The discussion does not provide enough context for why certain dietary patterns (e.g., high fat and sugar consumption) might prevail in Jordan, especially when exploring sex differences.
- Discuss the health implications of high fat and sugar consumption in detail, citing evidence from clinical or epidemiological studies. Highlight how Jordan is experiencing a nutritional transition and how this is reflected in the study’s findings.
- ‘Careful data quality control methods by reducing dietary intake misreporting’ was mentioned in the strengths section but not elaborated in methods section.
- How can you explain the representativeness of the dietary intakes at the individual levels based on 24hdr?
- The limitations section does not address other potential biases, such as recall bias from 24-hour dietary recalls or potential underrepresentation of dietary intakes.
- You have collected data on supplement use (yes/no), but the macronutrients intake assessment was based on foods and beverages consumed only without taking into consideration supplement use.
- The language is sometimes repetitive (e.g., repeated mentions of dietary guidelines and excess fat/sugar consumption).
- The structure could be improved by grouping related ideas more cohesively. Ideas presented in a somewhat scattered manner, making it harder to identify key points.
- While some recommendations are provided (e.g., controlling fat and sugar intake), they are vague and not strongly linked to the study’s findings.

Conclusions:

- The conclusion is overly focused on descriptive statistics and does not highlight the broader implications of these findings for public health or long-term health outcomes. It does not also include any recommendations for interventions, policy changes, or future research.

Is the work clearly and accurately presented and does it cite the current literature?

No

If applicable, is the statistical analysis and its interpretation appropriate?

Partly

Are all the source data underlying the results available to ensure full reproducibility?

Yes

Is the study design appropriate and is the work technically sound?

Yes

Are the conclusions drawn adequately supported by the results?

No

Are sufficient details of methods and analysis provided to allow replication by others?

Partly

Reviewer Expertise:

epidmeiology, biostatistics, maternal and child health, dietaty intakes

I confirm that I have read this submission and believe that I have an appropriate level of expertise to confirm that it is of an acceptable scientific standard, however I have significant reservations, as outlined above.

# Reviewer report: Reviewer response for version 2

**DOI:** 10.5256/f1000research.159480.r274011

**Published:** 2024-09-13

**Authors:** MoezAlIslam E. Faris

**Affiliations:** 1 University of Sharjah, Sharjah, Sharjah, United Arab Emirates

I was invited to review the manuscript titled: "Food consumption and adherence to dietary guidelines among Jordanian children and adolescents". The manuscript is important, tackles an interesting and timely issue in relation to the pandemic of obesity, especially . among children and adolescents. The manuscript is well-written, and relies on national validated data source. However, minor issues need to be adequately addressed to make this work more legible for publication. Forty (40) comments, edits, and suggestions need to be addressed, and are found in the attached PDF file.

 Pease find attached through the link:

*https://f1000research.s3.amazonaws.com/linked/675431.138866.pdf*

Is the work clearly and accurately presented and does it cite the current literature?

Yes

If applicable, is the statistical analysis and its interpretation appropriate?

I cannot comment. A qualified statistician is required.

Are all the source data underlying the results available to ensure full reproducibility?

Yes

Is the study design appropriate and is the work technically sound?

Yes

Are the conclusions drawn adequately supported by the results?

Yes

Are sufficient details of methods and analysis provided to allow replication by others?

Partly

Reviewer Expertise:

Nutrition, obesity, and inflammation

I confirm that I have read this submission and believe that I have an appropriate level of expertise to confirm that it is of an acceptable scientific standard, however I have significant reservations, as outlined above.

# Response

**Published:** 2024-09-14

**Authors:** Buthaina Alkhatib

Greetings 

 Thank you for the valuable notes and suggestions .

 All comments have been revised, and responses have been added to the comment boxes in the manuscript.

# Response

**Published:** 2024-09-20

**Authors:** Buthaina Alkhatib

response to reviewer  point by point: 

 https://doi.org/10.5281/zenodo.13810327

# Reviewer report: Reviewer response for version 2

**DOI:** 10.5256/f1000research.159480.r227951

**Published:** 2023-12-15

**Authors:** Lana Agraib

**Affiliations:** 1 Department of Food and Nutrition, Faculty of Agriculture, Jerash University, Jerash, Jerash Governorate, Jordan

No new comment

Is the work clearly and accurately presented and does it cite the current literature?

Yes

If applicable, is the statistical analysis and its interpretation appropriate?

Yes

Are all the source data underlying the results available to ensure full reproducibility?

Yes

Is the study design appropriate and is the work technically sound?

Yes

Are the conclusions drawn adequately supported by the results?

Yes

Are sufficient details of methods and analysis provided to allow replication by others?

Yes

Reviewer Expertise:

My areas of research are obesity, chronic disease, and dietary patterns

I confirm that I have read this submission and believe that I have an appropriate level of expertise to confirm that it is of an acceptable scientific standard.

# Reviewer report: Reviewer response for version 1

**DOI:** 10.5256/f1000research.152097.r223002

**Published:** 2023-11-27

**Authors:** Sarah Ibrahim

**Affiliations:** 1 Human Anatomy and Embryology Department, Tanta University, Tanta, Gharbia Governorate, Egypt

Thank you for submitting this paper. An interesting and important study appreciating the impact nutrition has on NCDs.

 Please find my feedback for some revisions below: 

**Abstract**

 The background is an articulation of the study purpose. Would be helpful to include some background information to substantiate the purpose of conducting this study- situate the problem first and then proceed to indicate the purpose of the study.

**Background**

 Perhaps including a little bit of information on the current overweight and/or obesity-related challenges in Jordan, that would have also sparked the interest to conduct this study.

**Methods**

 Reword: This study received ethical approval from the Institutional Board Review (IRB) committee, reviewed and approved the survey protocol at The Hashemite University (No.7/13/2020/2021). To: *Institutional Research Ethics Board approval was obtained from the Hashemite University (No.7/13/2020/2021).*

 Further clarification on where was data collected conducted?

 What type of sampling method was used? Further elaboration warranted.

 Rationale for excluding children with chronic disease warranted.

**Survey Tool**

 The following statement is unclear in this subsection: *It was announced on the university’s website about the need for nutritionists to work in data collection, and they were selected after personal interviews and based on their experience, and they were subsequently trained.*

 Recommend re-writing this section; as it stands, it is not entirely clear (semi-structured interviews, face-to-face interviews, various tools used)- particularly the first few sentences.

 Was the nutritionist present during the recall?

**Data Analysis**

 Should be written as stratified based on sex (and not gender).

**Discussion**

 Recommend condensing the discussion further (a number of studies are summarized here), and very little information provided on the implications section, which is important. Recommend highlighting key research articles in the discussion to substantiate your findings etc. and flush out the implications section further.

 A section on the limitations is warranted.

Is the work clearly and accurately presented and does it cite the current literature?

Yes

If applicable, is the statistical analysis and its interpretation appropriate?

Yes

Are all the source data underlying the results available to ensure full reproducibility?

Yes

Is the study design appropriate and is the work technically sound?

Yes

Are the conclusions drawn adequately supported by the results?

Yes

Are sufficient details of methods and analysis provided to allow replication by others?

Yes

Reviewer Expertise:

NA

I confirm that I have read this submission and believe that I have an appropriate level of expertise to confirm that it is of an acceptable scientific standard, however I have significant reservations, as outlined above.

# Response

**Published:** 2023-11-28

**Authors:** Buthaina Alkhatib

Thank you for submitting this paper. An interesting and important study appreciating the impact nutrition has on NCDs.

**Response: Many thnaks**

 Please find my feedback for some revisions below: 

**Abstract**

 The background is an articulation of the study purpose. Would be helpful to include some background information to substantiate the purpose of conducting this study- situate the problem first and then proceed to indicate the purpose of the study.

**Response: (It has been modified)**

**Background**

 Perhaps including a little bit of information on the current overweight and/or obesity-related challenges in Jordan, that would have also sparked the interest to conduct this study.

**Response: (It has been modified)**

**Methods**

 Reword: This study received ethical approval from the Institutional Board Review (IRB) committee, reviewed and approved the survey protocol at The Hashemite University (No.7/13/2020/2021). To: *Institutional Research Ethics Board approval was obtained from the Hashemite University (No.7/13/2020/2021).*

**Response: it has been modified**.

 Further clarification on where was data collected conducted?

**Response: it has been written in details in the section” study design and sample selection’, also the details were published in the protocol manuscript Al-Shami et al. (34)**

 What type of sampling method was used? Further elaboration is warranted.

**Response: simple random sampling for households. ( it has been provided** **)**

 The rationale for excluding children with chronic disease warranted.

**Response: the study aimed to investigate the children and adolescents in the whole apparently healthy population,  the exclusion of those with chronic diseases was** **to neutralize the effect of the disease on food intake and to reduce the effect of the medications taken on the dietary pattern and food consumption.**

**Survey Tool**

 The following statement is unclear in this subsection: *It was announced on the university’s website about the need for nutritionists to work in data collection, and they were selected after personal interviews and based on their experience, and they were subsequently trained.*

 Recommend re-writing this section; as it stands, it is not entirely clear (semi-structured interviews, face-to-face interviews, various tools used)- particularly the first few sentences.

**Response: “The university's website stated that nutritionists were required to assist with data collection. Based on their experience, they were chosen through in-person interviews, and they were then trained.”**

 Was the nutritionist present during the recall?

**Response: YES, for the first 24-hour recall, then the other recall was obtained by phone call. It has been clarified in the first 4 lines on the “dietary assessment” section**

**Data Analysis**

 Should be written as stratified based on sex (and not gender).

**Response** : **(It has been modified)**

**Discussion**

Recommend condensing the discussion further (a number of studies are summarized here), and very little information is provided on the implications section, which is important. Recommend highlighting key research articles in the discussion to substantiate your findings etc. and flush out the implications section further.

**Response: it has been modified and highlighted with red color.**

A section on the limitations is warranted.

**Response: it is available in the paragraph before the implications, and it has been separated into a single paragraph.**

# Reviewer report: Reviewer response for version 1

**DOI:** 10.5256/f1000research.152097.r204311

**Published:** 2023-09-12

**Authors:** Lana Agraib

**Affiliations:** 1 Department of Food and Nutrition, Faculty of Agriculture, Jerash University, Jerash, Jerash Governorate, Jordan

Thanks to the authors, this article is well-written and the figure and table are well-drawn and easier to read and understand helping in getting the idea more easily. The article addresses the food consumption pattern among Jordanian children and adolescents. It addresses an important topic in Jordan, especially indicating the consumed food group that may affect children and adolescents’ health, as the prevalence of obesity among this age group is rising dramatically.

 In more detail, Alkhatib and colleagues highlighted that the intakes of fruits, vegetables, and dairy products were low among the study group, while the consumption of additives specifically sugar and fat which was defined using the term "discretionary calorie" was high, and this may explain the increasing in obesity rate. It should make an important contribution to children and adolescent health programs which in consequence reduce obesity and the associated adulthood diseases that increase the burden in the health sector.

Is the work clearly and accurately presented and does it cite the current literature?

Yes

If applicable, is the statistical analysis and its interpretation appropriate?

Yes

Are all the source data underlying the results available to ensure full reproducibility?

Yes

Is the study design appropriate and is the work technically sound?

Yes

Are the conclusions drawn adequately supported by the results?

Yes

Are sufficient details of methods and analysis provided to allow replication by others?

Yes

Reviewer Expertise:

My areas of research are obesity, chronic disease, and dietary patterns

I confirm that I have read this submission and believe that I have an appropriate level of expertise to confirm that it is of an acceptable scientific standard.
